# Brief Myofascial Intervention Modulates Visual Event-Related Potential Response to Emotional Photographic Contents: A Pilot Study

**DOI:** 10.3390/vision7040077

**Published:** 2023-12-13

**Authors:** Gabriel Byczynski, Amedeo D’Angiulli

**Affiliations:** 1 Lab for Clinical and Integrative Neuroscience, Trinity College Institute for Neuroscience, School of Psychology, Trinity College Dublin, D02 PN40 Dublin, Ireland; byczynsg@tcd.ie; 2 Neuroscience of Cognition, Imagination and Emotion Research (NICER) Laboratory, Carleton University, Ottawa, ON K1S 5B6, Canada

**Keywords:** default mode network, salience, massage, ERP, parasympathetic, perception, emotion, touch

## Abstract

The use of touch for the treatment of psychiatric disorders is increasingly investigated, as it is shown that cognitive symptoms can be improved by various forms of massage. To investigate if the effect of massage is measurable using classical visual event-related potential components (P1, P2, late positive potential (LPP)), we performed a preliminary study on six participants using myofascial induction massage. Participants were shown emotionally valenced or neutral images before and after a 20 min myofascial massage. We found general increases in P2 amplitude following the intervention across all conditions (both neutral and affective), indicating increased attention or salience to visual stimuli. The magnitude of change was visibly larger for unpleasant stimuli, suggesting that visual perception and attention were modulated specifically in response to unpleasant visual images. The LPP showed reductions in amplitude after myofascial massage, suggesting increased emotional modulation following intervention, as a result of possible DMN alterations, consistent with region and function. We conclude that brief myofascial intervention supports other research in the field, finding that physical touch and massage techniques can alter cognition and perception. We posit further research to investigate its future use as an intervention for both physical and cognitive modulation. Importantly, we provide preliminary evidence that the neural processes that resonate with this type of massage involve complex feedforward and backward cortical pathways, of which a significant portion participate in modulating the visual perception of external stimuli.

## 1. Introduction

The effects of massage, or even simply physical touch, have been shown to elicit surprising effects on the body and nervous system. Massage has been investigated as a form of intervention in many domains, including pain [1,2], post-surgical comfort [3], migraines [4], and, most recently, for psychiatric disorders [5,6,7]. Various forms of massage have been explored as interventive therapies for psychiatric disorders, ranging from foot massage for improving symptoms in patients with schizophrenia, to Swedish massage for symptoms of generalized anxiety disorder, and general massage therapy for symptoms of anorexia nervosa. In many cases, these treatments have been shown to be effective in improving symptoms and quality of life in individuals, despite the mechanism(s) of action are still not completely understood.

One often-cited possible account for the improvements associated with massage interventions may be the nervous system cascade effects resulting from the activation of the parasympathetic nervous system (PNS). It has been shown that markers of autonomic nervous system activation typically correspond to moderate pressure massage, suggesting increases in vagal efferent activity [8]. These changes are believed to originate from the stimulation of pressure receptors in the skin, an effect that has been demonstrated in the literature (e.g., see [9]). Activation of the PNS, a system that complements the fight or flight response from the sympathetic nervous system (SNS), results in reductions in heart rate and blood pressure. The PNS is, thus, an attractive target when attempting to counteract the effect of SNS arousal and modulate emotional responses, for example. Contribution to the activation of the PNS does not only come from the vagus nerve, but also from the oculomotor, facial, and glossopharyngeal nerves [10]. In one study, it was shown that by massaging the vagus nerve, PNS activity increased, while subjective stress levels decreased. Heart-rate variability, a marker of PNS activity, increased only in the massage groups compared to the control group [11]. Accordingly, there is a clear connection between touch-based interventions such as massage and the modulation of the PNS, either via direct stimulation of the cranial nerves or via the effect of physical touch in general. 

In early research using EEG [12], 26 participants received a chair massage, while 24 control group adults were asked to relax in the massage chair for 15 min, twice a week, for five weeks. EEG was performed before, during, and after the first and last sessions. In addition, before and after the sessions, participants performed math computations, completed depression and anxiety scales, and provided saliva for cortisol analysis. The results of the study revealed that frontal delta power increased for both groups, indicative of general relaxation. The massage group showed decreased frontal alpha and beta power, indicating enhanced alertness, while the control group showed increased alpha and beta power. Lastly, the massage group showed improved speed and accuracy on math computations, while the control group did not show changes. Anxiety levels were lower following massage, but not after the control sessions, although both groups showed an improved mood state. Salivary cortisol levels were lower following the massage, but only on the first day, and at the end of the five-week period, depression scores were lower for both groups, with job stress scores lower for only the massage group. 

In two follow-up studies, a sample of depressed adolescents received a 15 min chair massage, resulting in lower right frontal EEG activation, known to be involved in negative affect and withdrawal tendencies [13]. A similar pattern of right frontal EEG asymmetry was found in one-month-old infants of depressed mothers [14], supporting the hypothesis that massage involves changes in PFC asymmetry related to emotional processing.

Further, in a recent fMRI study [15], healthy participants were randomly assigned to one of four conditions: Swedish massage, reflexology, massage with an object, or a resting control condition. The right foot was massaged while each participant performed a cognitive association task in the scanner. Results showed that the Swedish massage treatment activated the subgenual anterior and retrosplenial/posterior cingulate cortices. This increased blood oxygen level dependent (BOLD) signal was maintained only in the former brain region during the performance of the cognitive task. The reflexology massage condition selectively affected the retrosplenial/posterior cingulate in the resting state, whereas massage with the object altered the BOLD response in this region during the cognitive task. The authors interpreted the results as showing that massage modulates DMN regions and influences arousal and conscious attention, especially because of the known pleasant affective properties of touch. 

More recently, the research focus expanded to the effects of massage on functionality related to arousal alterations, which mediate changes in voluntary conscious attention, such as the effect on the default-mode network (DMN) and the inversely correlated Attentional/Task Positive Network (ATPN). A PET study [16] involving healthy participants revealed that back massage lasting 4 min increased cerebral blood flow in the left precuneus and pons, with an increase evident in bilateral precuneus and left fusiform gyrus after 20 min of further massage. These effects were also correlated with changes in the amygdala, basal forebrain, and hypothalamus, and increased parasympathetic activity (i.e., heart-rate variability). 

In another fMRI study by Strauss and colleagues [17], it was found that physical touch produced increases in blood oxygen level dependent (BOLD) responses in the secondary somatosensory cortex, superior temporal gyrus, and the primary somatosensory cortex. This finding was highlighted by the fact that the BOLD response was more pronounced during the interpersonal touch intervention, as compared to the impersonal touch intervention, suggesting that the effect induced is not strictly touch-related, but it is also context-dependent. 

Thus, there is a growing body of evidence that frames massage, a form of physical touch, as a modulator of not only PNS activation, but possibly also as one that can alter DMN activity via affective responses. It may be that massage offers a method of modulating more than pain, anxiety, or psychosomatic disorders. Specifically, massage may influence healthy brain network activity related to complex higher cortical or “top-down” cognitive functions [18] such as visual perception. Vision entails complex high-level processes involving both forward and backward input from complementary cortical streams of processing, beyond the visual areas [19]. The perception of complex visual contents such as emotional images, in particular, can engage parts of the frontal cortex, limbic regions, and amygdala to elicit adaptive response and possible subsequent decision making and/or action [20]. In particular, images containing evident emotional connotation, such as deeply unpleasant, i.e., bodily mutilation, or very pleasant images, i.e., smiling newborn, often can recruit regions such as the prefrontal cortex [21]. 

### 1.1. The Present Study

We aimed to determine how a particular massage technique, myofascial induction massage therapy, known for its anxiolytic properties [22,23,24] may alter the activation of the DMN and, as a result, may have an influence on the visual processing of complex photographic images with emotional contents. Myofascial induction massage therapy (MIMT) usually involves a trained massage therapist depressing the muscle and surrounding connective tissue to induce a release of muscle tension. This spontaneous release of tension may activate the parasympathetic nervous system and, in turn, promote relaxation which might promote a generalized modulation of perception of stimuli presented in any sensory modality [25], including visual stimuli. Therefore, a plausible general hypothesis is that MIMT might influence emotional salience and attention associated with visual stimuli. 

Event-related potential (ERP) techniques provide an objective and well-established measure of the effects of salience and attention by focusing on the enhancement of the early components of the visual ERP such as the P1, N1, and P2. These components would thus represent active attentive states, compared to passive or perceptive states [26,27]. Relaxed states have been historically linked with attention, such that relaxation promotes attentiveness, cognition, and emotional regulation [28]. Emotional salience of images also changes the attentive state, as it has been shown that emotionally charged images amplify attention, as measured by ERP amplitude (see [29]). Late potentials such as the late positive potential (LPP) are also known to be markers of emotional salience, as the LPP in the posterior electrodes is shown to increase during exposure to unpleasant images [30]. Given the ethical issues involved in displaying visually graphic and potentially traumatic contents, we used a person-level approach with “professional observers” as participants [31] to assess whether the effects of MIMT and massage therapies on visual perception could be reliably detected by using the small-N designs often used in psychophysics and vision research [27]. 

Approaching research at the individual level implies that repeated-measures EEG features have an acceptable precision within the individual (e.g., *n* = 1) [31,32]. Furthermore, EEG personalization faces issues that overlap with the inherent debate between measuring person-oriented approaches [33,34] and the population-distribution referenced approach (for further discussion, see [35]). Personalized prediction requires prior data, and is most often biased by the addition of aggregated data from a population [36]. This occurrence is largely due to assumptions made by classical ergodicity theorems [37], in which intraindividual and interindividual variations are equivalent. Where intraindividual variations are nonhomogenous, applying equivalence to interindividual variation becomes inappropriate. The nonergodic implications therefore apply to functional brain connectivity [38,39] and neural network dynamics (e.g., see [40]) notably as reflected by measured ERP patterns and dynamics. Employing small-N designs that investigate the individual as the replication unit and the control of themselves is therefore a valid alternative and/or supplementary tool to assess EEG variations that may be individually heterogeneous. Alternative approaches conforming to traditional experimental designs do exist [32,41], one being the method of the vincentized averaging [42]. In this technique, small sample data are binned; thus, the resulting distribution average from the bins represents a reconstructed distribution with a shape that does not deviate too much from the one displayed by the distribution of observations from each individual subject. 

In the foregoing experiment, participants were shown a series of unpleasant, neutral, and pleasant real-life photographs from the International Affective Picture System (IAPS) [43] before and after a 15 min MIMT. The task involved active visual perception requiring participants to study the photos for a subsequent visual mental imagery test. ERPs were recorded pre- and post-MIMT throughout the experiment. We displayed images with emotional content to determine how MIMT might alter the perception of stimuli, and to explore the possible changes in the resulting ERPs, specifically the early visual components (P1–P2) and late positive potentials (LPPs) as a measure of the hypothesized high-order-modulating action of MIMT. 

Based on the literature reviewed, we predicted early visual ERP components (P1–P2) to be heightened across the scalp, but especially in relation to the somatosensory and posterior, occipital, and parietal cortices. We further predicted that the P1–P2 components associated with emotional salience, i.e., pleasant or unpleasant stimuli, should be amplified relatively more than in association with neutral content. In contrast, we predicted that, due to the active nature of the perceptual task requiring the encoding of the visual contents in working memory (as a byproduct of the task demand of preparing to generate visual mental images), the LPP response would show a sustained enhancement in association with neutral and pleasant photos but a reduction in association with unpleasant photos; temporally, we expected the latter pattern to be evident first in frontal electrodes and then posteriorly.

## 2. Materials and Methods

### 2.1. Participants

The data were collected during repeated long recording session samples from “professional observers” in the span of 48 h. Participants were six healthy university graduate students (mean age 24.66 years). All participants provided verbal consent to participate and waived the requirement of signed consent. The study was part of a grant-funded pilot project which was retrospectively assessed and approved by Carleton University Institutional Research Ethics Board (section Human Subjects) under the regulation set by the Canadian Tri-Council. Participants were compensated with course credits for a graduate seminar course. 

All participants were right-handed males with corrected-to-normal or normal vision, and none reported any history of neurological impairment or were currently using psychoactive medications. The average number of hours of sleep on the previous night was 7.5 h. Testing was conducted from 10 a.m. to 4 p.m., with a one-hour break in between on two separate days. All participants completed the Beck Depression Inventory (BDI) and State Trait Anxiety Inventory (STAI), with exclusion criteria set at 15< or 60<, respectively. Participants did not declare previously diagnosed mental or neurological disorders. In addition, the pre-experimental short adult version of the mood and feelings questionnaire [44] was administered by an independent research assistant unaware of the hypotheses and goals of the study to screen for mood differences or emotional changes before the experimental sessions between days. No differences were reported, and all participants scored similarly (overall score range: 3–5) over the two days. These scores were below the recommended clinical cut-off (possible maximum = 26; clinical cut-off ≥ 11). Prior to the experiment, all participants gained familiarity with the real stimuli and conditions of the tasks during several study sessions. Participants were involved in selecting the stimuli and the conditions, they designed the computer programs for the tasks, and they piloted the delivery and the performance of the task computer programs and instrumentation. Given that these activities involved extensive exposure and practice over time, it can be assumed that the materials of the tasks were overlearned and practice effects, particularly due to the order in which the devices were used, were no longer present. This can be considered as compensation for lack of counterbalancing due to small sample.

### 2.2. Procedure

The procedure was adapted from the previously published experimental protocol by Marmolejo-Ramos et al. [29]. The task involved two active visual perception phases interjected by a 20 min complete standard myofascial manipulation intervention. The repeated measures experimental design included three phases: (1) pre-EEG recording coupled with an International Affective Picture System (IAPS) perception task with the commission of visual mental image generation; (2) a myofascial induction massage (MIMT); and (3) a post-EEG recording coupled again with the IAPS perception task. 

Participants’ EEGs were recorded with EEG “quick-caps” with silver chloride electrodes (Neurosoft, Inc., Sterling, VA, USA). Each participant had 32 Ag–AgCl electrodes applied according to the 10–20 international system. All electrodes were referenced to a separate electrode located on the nose tip with AFz ground. Impedances were kept and continuously rechecked to be below 5 kOhms. The vertical electrooculograms (VEOG) were recorded from two split bipolar electrodes on the left and right supraorbital ridges (VEOGU, L and R) as well as the left and right zygomatic archs (VEOG, L and R). The signal from the electrodes was amplified and digitized by a SynAmps2 and a SCAN™ 4.3 EEG system (Compumedics Neuroscan, El Paso, TX, USA) with filter settings at 0.15 Hz (high pass) and 100 Hz (low pass). The data were digitized online at a sampling rate of 1000 Hz. Nine channels were used: Oz, Pz, Cz, F4, FC4, F3, FC3, Fz, and VEOG, following previous work which identified these as critical electrodes of interest for visual processing [45,46]. Ten practice trials (neutral imagery) ensured instruction comprehension. The task was carried out in three phases. During the first IAPS perception task, participants were exposed to 300 total images subdivided in blocks of 20 photos all containing neutral, pleasant, or unpleasant contents; these “thematic” blocks, however, were presented in random order. Participants were engaged in the IAPS task while EEG was concurrently recorded. Each picture was displayed for 10 s, followed by a blank screen which lasted for 4 s. We focused only on the initial 1000 ms following image onset for perception, which, as shown by previous work, provides enough time to capture the correlates of perception without overlapping into the imagery phase that follows [47,48]. 

**Figure 1:**
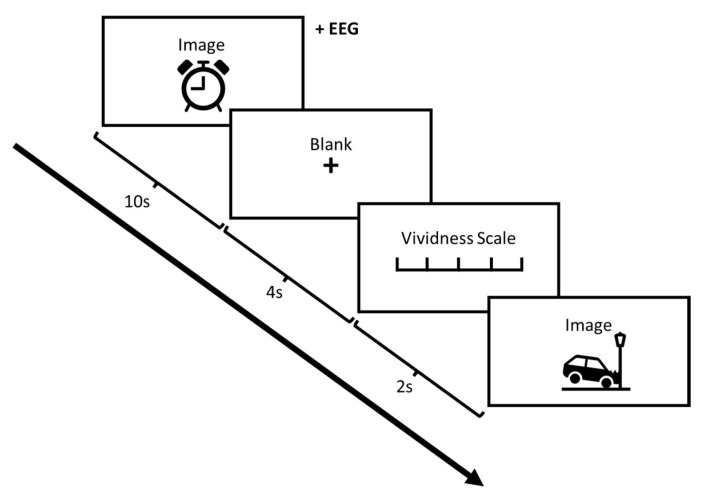
Schematic of image presentation and trial structure.

Participants were asked to study each photo and then generate a visual mental image of the contents they had just viewed. The task of generating a subsequent visual mental image was to make sure that the participants engaged in active perception even if they were not required to respond. Indeed, to enforce this aspect, the participants were asked to keep in mind that the experimenter could ask details about the imagined content after each trial. In the present paper, only the perception phase is considered, and data pertinent to the imagery phase will be the basis of another foregoing follow-up study. The ERP was recorded with onset at the start of each trial. The participant was then asked to report the vividness of his/her image via a Likert scale from 1–5. After 2 s, the next photo was displayed. In phase 2 of the experiment, the participant received a 20 min MIMT treatment while lying prone (see Section 2.3). In phase 3, the participant repeated the same procedure as phase 1. Two sets of buffering neutral images were presented to minimize confounds of unpleasant/pleasant content [49]. After each experimental phases 1 and 2, participants were shown 20 pleasant photos to counteract possible negative emotions following unpleasant stimuli. Although we aimed to address the possibility of emotional carry-over effects with the interspersion of neutral trials and the counteraction with pleasant photos, this does not remove the possibility of the effect. Thus, our results should be taken in this context. This procedure schematic is presented in Figure 1. To control for mind-wandering and its effects, we asked the participants to focus on a specific structured imagery task, a method known to engage specific imagery behavioural mechanisms [50,51]. In brief, this task requires the participant to project the remembered image onto the blank screen from a frontal perspective as a 2D picture, and to view their imagery as “complete” if they cannot increase detail.

### 2.3. Myofascial Intervention

The MIMT was carried out following four standard techniques outlined in [52]. In brief, the four techniques targeted the cervical segment, shoulder girdle, brachial, and ante-brachial fascia. Accordingly, participants received sub-occipital, suprahyoid, scalene muscles, and prevertebral fascial induction in the cervical segments. In the shoulder girdle: induction of the pectoralis, deltoid, clavi-pectoral, arm, trapezius, and spine of the scapula, and in the brachial and ante-brachial techniques: telescopic three-dimensional induction and interosseous induction. More detailed procedures can be found in [53]. The intervention was delivered by an experienced certified physiotherapist specialized in MIMT techniques who did not know the details of the hypotheses and was not familiar with cognitive neuroscience or psychology or EEG/ERP techniques.

### 2.4. Stimuli

Photos were selected from the IAPS [43]. Pleasant, unpleasant, and neutral images were selected based on normative IAPS valence scores, with categories containing images rated in the top 20%, bottom 20%, and middle 20%, respectively. In both perception tasks used in the present experiment, 100 of each were shown, for a total of 600 total stimuli. The second set of unpleasant images, shown in stage 3, were objectively (i.e., by ERP amplitude) and subjectively more stressful than in stage 1 (as shown in a previous study [42]), to counteract any habituation effect. The characteristics of the selected stimuli are presented in Table 1.

### 2.5. Data Analysis

Participants’ data were vincentized (partitioned according to time-series bins with same data density per interval of time by averaging the participants’ quantile functions) [54,55]. Successively, bin-by-bin means were estimated for each electrode, ensuring the definition of group quantiles, constructing a distribution function for each electrode. The EEG data were analysed using BESA 5.2 and MATLAB R2011, with a filtering cutoff of 20Hz [56], 0 phase, and slope of 12db/oct. Artifacts from eye movement were eliminated via artifact scanning. A total of 20 trials from each emotion condition from both stage 1 and 3 (pre- and post-treatment) were averaged into −200 ms to 1000 ms epochs. ERP data traces were binned in 30 ms intervals, following the vincentization approach (see Section 1.1, and [42]), using IBM SPSS prior to analysis, and smoothed in Python using SciPy interpolation, k = 3.

Significance for planned t-test comparisons was based on the computed average family false discovery rate (FDR) threshold.

## 3. Results

### 3.1. P1–P2 and LLP

**Figure 2:**
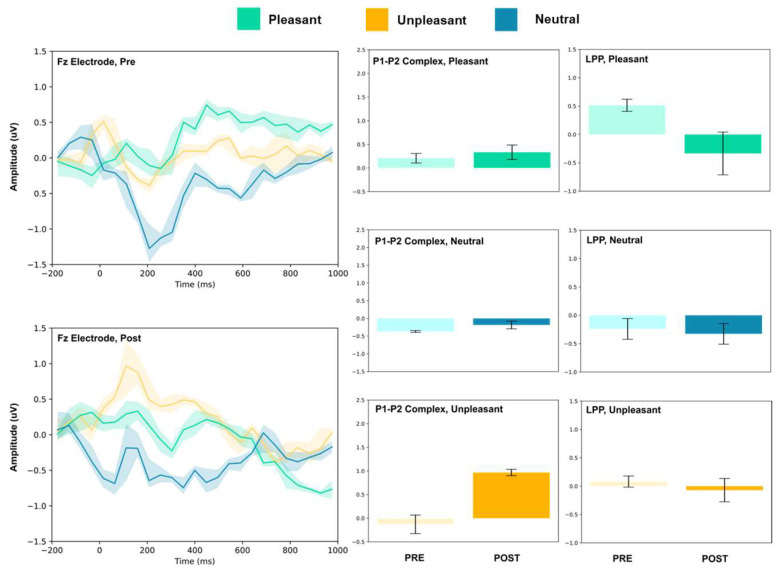
Fz electrode ERP for pleasant, unpleasant, and neutral visual stimuli (**left**). P1–P2 complex (**middle**) and LPP (**right**) for each condition (pre and post). Error bars represent ± 1 standard deviations. Nonoverlapping bars represent significance at *p* < 0.028.

**Figure 3:**
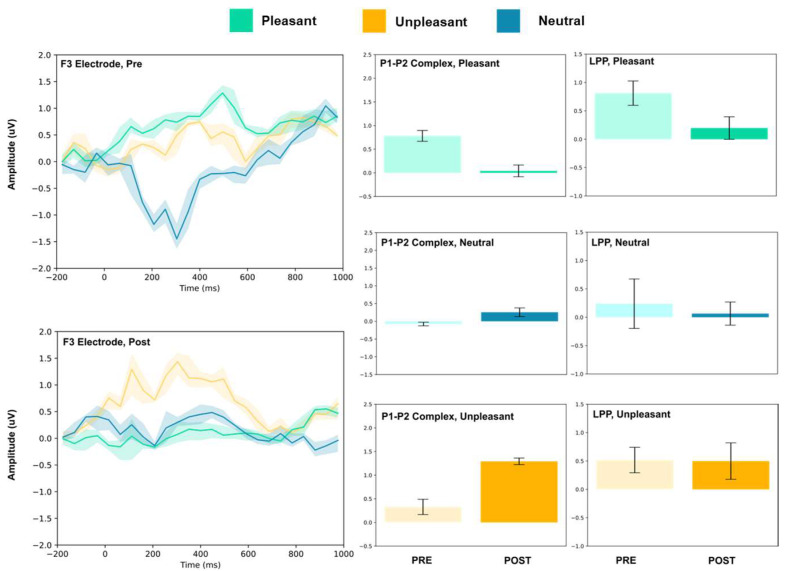
F3 electrode ERP for pleasant, unpleasant, and neutral visual stimuli (**left**). P1–P2 complex (**middle**) and LPP (**right**) for each condition (pre and post). Error bars represent ± 1 standard deviations. Nonoverlapping bars represent significance at *p* < 0.028.

**Figure 4:**
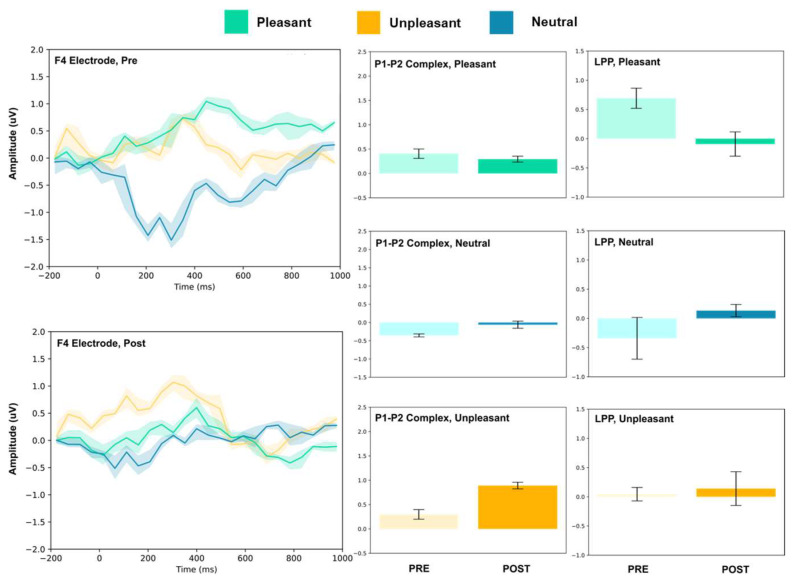
F4 electrode ERP for pleasant, unpleasant, and neutral visual stimuli (**left**). P1–P2 complex (**middle**) and LPP (**right**) for each condition (pre and post). Error bars represent ± 1 standard deviations. Nonoverlapping bars represent significance at *p* < 0.028.

**Figure 5:**
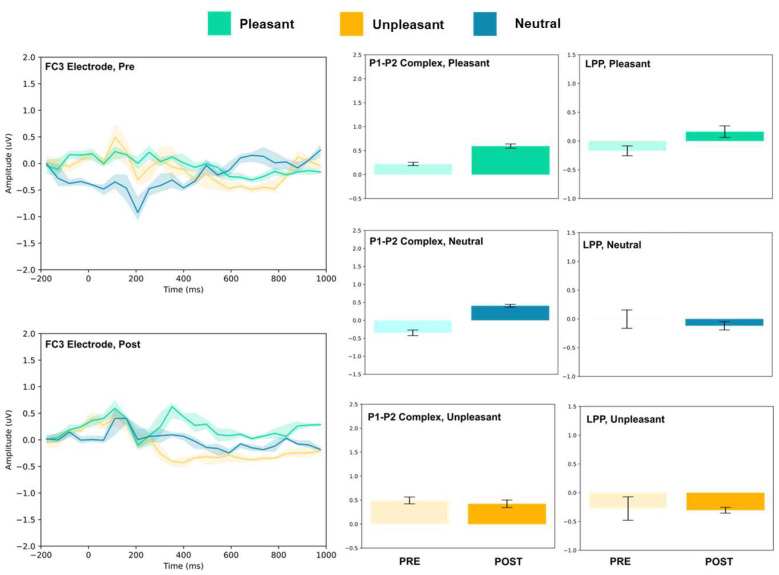
FC3 electrode ERP for pleasant, unpleasant, and neutral visual stimuli (**left**). P1–P2 complex (**middle**) and LPP (**right**) for each condition (pre and post). Error bars represent ± 1 standard deviations. Nonoverlapping bars represent significance at *p* < 0.028.

**Figure 6:**
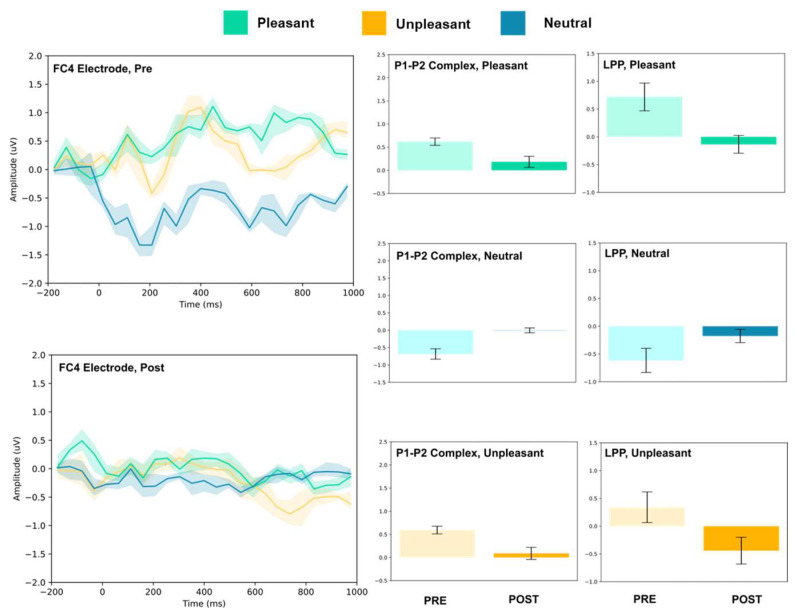
FC4 electrode ERP for pleasant, unpleasant, and neutral visual stimuli (**left**). P1–P2 complex (**middle**) and LPP (**right**) for each condition (pre and post). Error bars represent ± 1 standard deviations. Nonoverlapping bars represent significance at *p* < 0.028.

**Figure 7:**
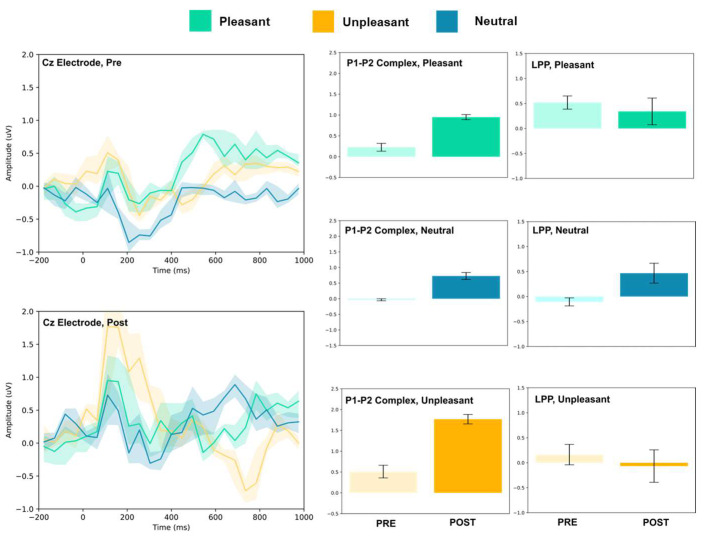
Cz electrode ERP for pleasant, unpleasant, and neutral visual stimuli (**left**). P1–P2 complex (**middle**) and LPP (**right**) for each condition (pre and post). Error bars represent ± 1 standard deviations. Nonoverlapping bars represent significance at *p* < 0.028.

**Figure 8:**
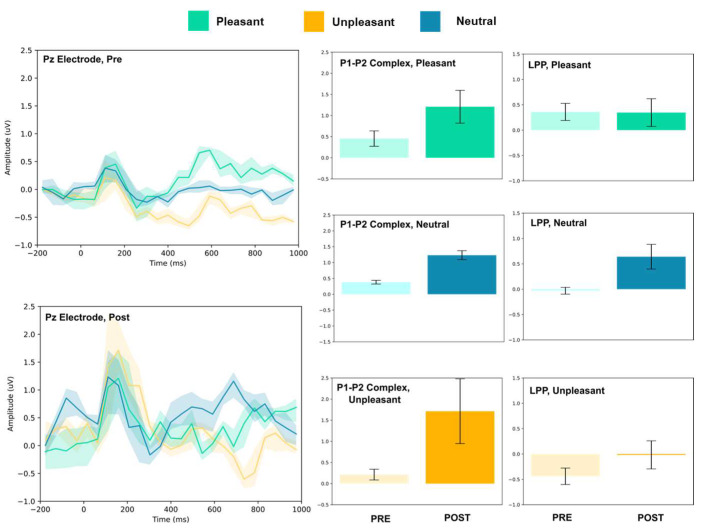
Pz electrode ERP for pleasant, unpleasant, and neutral visual stimuli (**left**). P1–P2 complex (**middle**) and LPP (**right**) for each condition (pre and post). Error bars represent ± 1 standard deviations. Nonoverlapping bars represent significance at *p* < 0.028.

**Figure 9:**
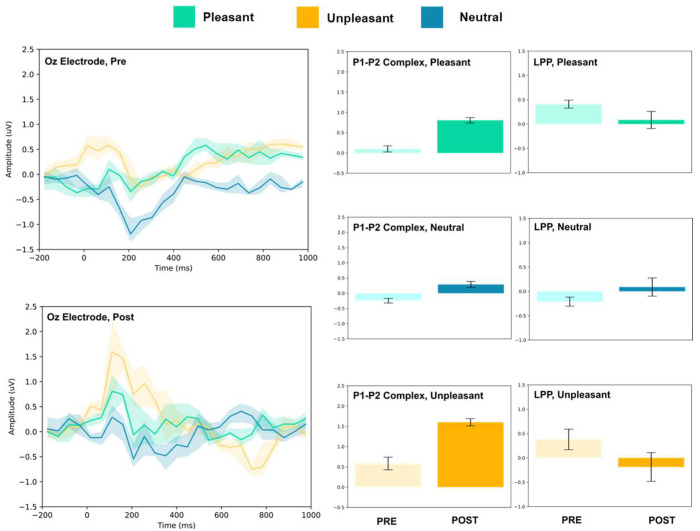
Oz electrode ERP for pleasant, unpleasant, and neutral visual stimuli (**left**). P1–P2 complex (**middle**) and LPP (**right**) for each condition (pre and post). Error bars represent ± 1 standard deviations. Nonoverlapping bars represent significance at *p* < 0.028.

The amplitude of the P1–P2 complex before and after MIMT intervention was measured in response to unpleasant, pleasant, and neutral stimuli. Results for each electrode, including the ERP traces for all conditions, are presented in Figure 2, Figure 3, Figure 4, Figure 5, Figure 6, Figure 7, Figure 8 and Figure 9. A *t*-test was performed on the pre/post-amplitudes, and for clarity the results are presented in Table 2. The mean late-positive potential (LLP) was defined visually as starting at 450 ms post-stimulus, in line with previous work estimating emotion-induced LPP, and continuing for approximately 1000 ms [57]. The results of the LPP are presented alongside the P1–P2 complex in Figure 1, Figure 2, Figure 3, Figure 4, Figure 5, Figure 6, Figure 7 and Figure 8 and in Table 2.

#### 3.1.1. Frontal Electrodes

The frontal electrodes (Fz, F3, and F4) consistently showed increased P1–P2 amplitude for unpleasant images. While the LPP decreased following intervention at F3, it increased at Fz and remained unchanged at F4. For neutral stimuli, there was a general increase of the P1–P2 component following intervention; however, only the F3 electrode showed a change (decrease) in amplitude. For pleasant stimuli, the F3 and F4 electrodes showed a general decrease in P1–P2 amplitude and LPP, while the Fz electrode showed increases following intervention.

#### 3.1.2. Frontocentral and Central Electrodes

The FC3, FC4, and Cz electrodes showed an overall increase in P1–P2 amplitude to unpleasant images, and the Cz and FC4 electrodes specifically showed a decrease in LPP. For neutral stimuli, all of FC3, FC4, and Cz showed increased P2 amplitude, and all except FC4 showed increased P1 amplitude. LPP also increased for FC4 and Cz; however, it decreased for FC3. Lastly, for the pleasant images, all three electrodes showed increased P2 amplitude, and all except FC4 showed increased P1 amplitude. FC3 showed increased LPP, FC4 showed decreased LPP, and no change was observed for the LPP at Cz.

#### 3.1.3. Parietal and Occipital Electrodes

At Pz and Oz, all stimuli evoked larger amplitude P1–P2 responses. Increased LPP was observed for neutral stimuli at both sites, and decreased LPP was observed at Oz for pleasant and unpleasant stimuli. No change in LPP was detected at Pz for either stimulus.

## 4. Discussion

**Figure 10:**
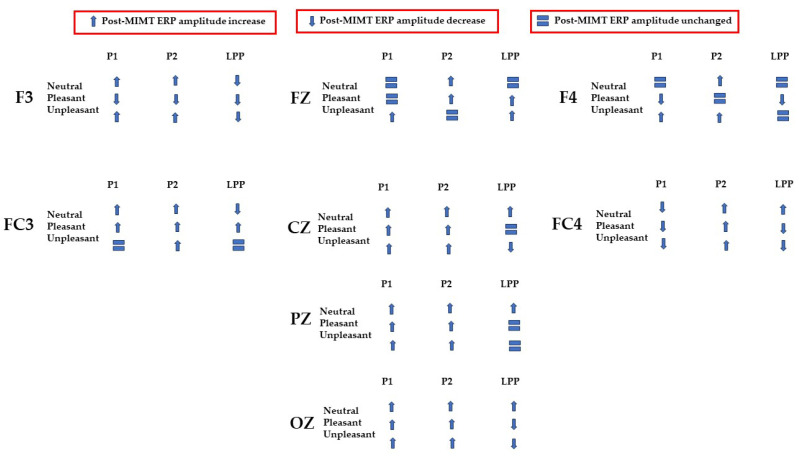
Summary of ERP amplitude changes (increases represented as upward arrows; decreases represented as downward arrows) or null effects (represented as equal signs) post-MIMT intervention.

In the present study, we investigated the effect of MIMT on electrophysiological correlates of salience, visual processing, and attention with the aim of understanding how MIMT may influence the perception of emotionally valenced and neutral images. A summary of the complex pattern of results is given in Figure 10.

Early ERP components (<300 ms) have been linked with attention to unpleasant pictures, and affective stimuli primarily modulate the amplitude of these components [58,59]. Thus, we may interpret P1–P2 amplitude changes after MIMT intervention as an alteration of attention to images. Our results show that, generally, the P1–P2 amplitude significantly differed pre- to post-MIMT. Frontally (Fz, F3, F4), there was an increase in P1–P2 complex amplitude for the neutral and unpleasant visual stimuli, and a decrease in amplitude for pleasant stimuli (F3 and F4 only). The magnitude of change from pre- to post-intervention also appeared most prominently for unpleasant stimuli, which corroborates the hypothesis that, typically, early components such as P1 and P2 are modulated by affective stimuli and are linked to attention to unpleasant pictures. In this context, an increase in the frontal amplitude, which is most prominent for unpleasant stimuli, suggests that MIMT intervention may modulate attention to affective stimuli. 

Moving from frontal to more posterior electrode sites, there was an even greater magnitude of change for the P1–P2 complex for the unpleasant stimuli (peaking at Cz). Indeed, it has been shown that the posterior P2 amplitude increases with general attention or perception, an effect which was additionally noted frontally (frontal eye field) [60]. Therefore, our findings showed a general posterior increase in the P1–P2 amplitude across all conditions, which we may attribute to MIMT-related increases in attention. Consistency across all conditions, including the neutral visual stimuli, indicates that the increase in amplitude reflects general attention or salience, which is not restricted to affective stimuli specifically. 

Frontal increases in P1–P2 amplitude that show greater magnitude comparatively indicates that unpleasant stimuli produce larger changes in P1–P2 amplitude following MIMT, thereby suggesting a more specific modulation for affective stimuli, similar to those mentioned previously [58,59]. In the LPP, we also found patterns of decrease in mean amplitude between pre- and post-intervention. Frontally, there was a decrease in LPP amplitude for pleasant visual stimuli, and at FC3 and Oz, both pleasant and unpleasant stimuli had decreases in LPP. One interpretation is that the decreased LPP may represent enhanced emotional regulation, as it has been shown that suppressing emotional responses correlates with reduced LPPs [61]. This finding would complement the theory that MIMT alters (frontal) DMN activation, subsequently altering emotional regulation [62]. 

Taken together, our findings suggest that MIMT may modulate processing of visual contents via general salience, affect-specific attention, and emotional regulation. Possible routes have been suggested in previous research and in the literature, including some mentioned here (PNS, vagal regulation), but also those including endocrine or immune regulation [63]. As previous work has shown that interpersonal touch and massage activates numerous areas of the brain, switching SNS to PNS dominance in animals, and increasing PNS activity in humans, we suggest that the resulting changes in perception and attention may reflect vagus- or PNS-induced changes to visual perception and attention (as also reported in [11,17,64]).

**Figure 11:**
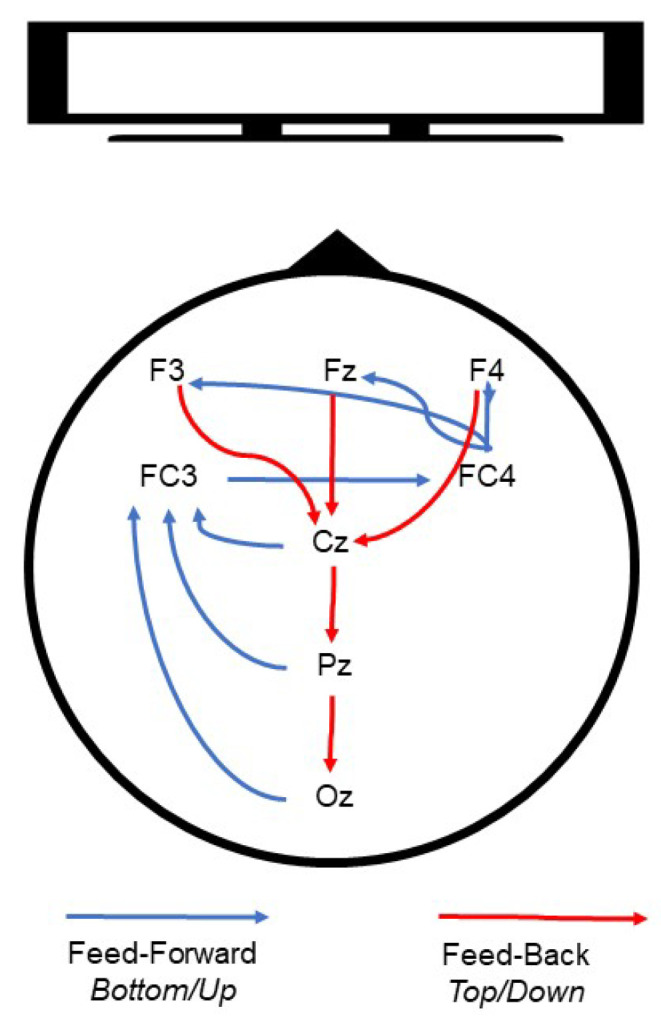
Schematic representation of cortical dynamics during visual processing.

This study contributes to the understanding of the interplay between feedforward and feedback cortical dynamics of visual processing and perception, and the neural networks involved in emotional regulation and salience. The larger picture our results suggest is one that integrates the roles of left and right, and frontal and occipital regions, in a (potentially cyclic) network of modulation in line with the other networks which are posited to be involved in emotional regulation [65] and affective processing [66]. We suggest a graphic synthetic interpretation in Figure 11, following the flow of information as reflected in Figure 10. 

Visual information may be initially processed in the occipital regions (Oz) and subsequently encoded and retained in frontal areas including the lateral prefrontal cortices (LPFC), regionally similar to electrode FC3 [67]. Simultaneously, based on previous work, we propose that it is possible that the right hemisphere (FC4) processes visual specifics, while the left hemisphere processes more general and conceptual object features [68]. Interestingly, the FC3 and FC4 electrodes have been considered an approximate location of the frontal eye fields (left and right, respectively) [69], which have been attributed to visual attention modulation [20]. 

After the initial stage, valenced by attention, visual information would then be processed by higher-order frontal regions (F3, Fz, F4), regions that have been associated with control and regulation of the valence of emotional experiences [66]. These bottom-up or feedforward dynamics are then complemented by feedback processes which we suggest may be top-down and may have the role of modulating visual processing. In this instance, frontal regions could modulate through downward-cascade visual attention in more caudal regions (Oz), creating looping cortical dynamics which are updated based on emotional information and subsequent processing [70]. Although we recognize that this model is hypothetical, we believe that the current literature and our findings combined support this interpretation. Specifically, location-specific involvement (i.e., frontal visual information processing, central encoding, and initial occipital processing) and inter-regional interaction (i.e., frontal–central–occipital processing circuitry), which are supported by previous work, fit with our proposed hypothetical model and the complete pattern of data, as shown in Figure 10.

Through MIMT intervention, we suggest that aspects of the abovementioned dynamics are altered, resulting in heightened attention/salience to images, and increased emotional processing, particularly to emotionally stressful images. For clarity, we present schematics of this hypothetical model (capturing flow of information during pre- and post-intervention) in Figure 11. The specific mechanisms that would be responsible for modulating these bottom-up and top-down dynamics need to be detailed in future research. In particular, to verify this hypothetical model, source localization and Granger causality analysis on a higher-density montage would be required to draw more precise locational and dynamical conclusions about connectivity and information direction. We also refrain from discussing possible critical bottom-up influences of subcortical regions such as the amygdala but recognize that they likely contribute to the networks suggested here. To disentangle these complex set of contributions, future research should employ a combination of EEG and fMRI techniques. 

Since this was an initial pilot study, our findings are restricted to the (all male) sample that was analysed. Thus, our results are currently limited to interpretation in this context. Sex- or gender-based differences may arise in visual processing to emotional stimuli, as has been reported previously [71]. Consequently, future research should use a gender- and age-diverse sample to uncover any generalizable findings surrounding the effect of MIMT on visual processing, specifically with more in-depth analyses allowing for causative and temporally and spatially informative findings.

## 5. Conclusions

We reported a small-n study highlighting the ERP changes following MIMT intervention using a visual perception task. Our findings highlight two key points: (1) that small-n studies can indeed be utilized for the investigation and measurement of personalized or within-individual changes to perceptual ERP correlates, and (2) that MIMT fits in with the growing literature showing that interventions such as massage can impact cognition, in this instance, in the form of perception and attention. Here, we showed that P2 and LLP ERP features are altered following MIMT, suggesting increased attention, increased emotional perception, and further suggesting possible alterations to the DMN network functioning. This suggests that massage techniques, such as brief myofascial induction, can measurably alter correlates of visual attention, justifying further research to investigate their potential use as a modulator of affective response.

## Figures and Tables

**Table 1:** Arousal and valence normed values of the IAPS photos used in the two phases (pre- and post-myofascial induction massage therapy, MIMT) of the experiment.

	Pre-MIMT	Post-MIMT	
			T(198) *
	Arousal		
Neutral	3.2 (0.3)	3.5 (0.5)	4.68
Pleasant	5.8 (0.4)	6.1 (0.2)	6.71
Unpleasant	6.8 (0.2)	6.9 (0.1)	4.47
	Valence		
Neutral	5.2 (0.1)	5.4 (0.3)	6.32
Pleasant	7.6 (0.3)	7.8 (0.4)	4.00
Unpleasant	2.4 (0.5)	2.8 (0.6)	5.12

Note. * All *p* < 0.0001.

**Table 2:** Pre- and post-intervention P1–P2 complex and LPP for each electrode across all conditions.

Condition	Electrode	P1–P2 Amplitude (uV)		T(5)	LPP (Mean uV)		T(5)
		Pre ± SD	Post ± SD		Pre ± SD	Post ± SD	
Pleasant	Fz	0.21 ± 0.10	0.33 ± 0.15	1.67	0.51 ± 0.11	−0.33 ± 0.38	5.31 ***
	F3	0.78 ± 0.11	0.04 ± 0.12	10.72 ***	0.81 ± 0.21	0.19 ± 0.20	5.15 ***
	F4	0.41 ± 0.10	0.29 ± 0.06	2.40	0.69 ± 0.17	−0.09 ± 0.21	7.11 ***
	FC3	0.22 ± 0.03	0.59 ± 0.04	16.10 ***	−0.17 ± 0.09	0.16 ± 0.10	6.16 ***
	FC4	0.62 ± 0.08	0.18 ± 0.11	7.45 ***	0.72 ± 0.25	−0.13 ± 0.16	7.05 ***
	Cz	0.22 ± 0.10	0.95 ± 0.06	15.75 ***	0.52 ± 0.13	0.34 ± 0.27	1.46
	Pz	0.45 ± 0.18	1.21 ± 0.39	4.31 **	0.36 ± 0.17	0.35 ± 0.27	0.10
	Oz	0.10 ± 0.08	0.81 ± 0.07	17.02 ***	0.41 ± 0.08	0.08 ± 0.17	4.10 **
Neutral	Fz	−0.37 ± 0.03	−0.18 ± 0.11	4.01 **	−0.24 ± 0.18	−0.33 ± 0.18	0.82
	F3	−0.07 ± 0.05	0.25 ± 0.12	6.11 ***	0.24 ± 0.44	0.06 ± 0.20	0.88
	F4	−0.35 ± 0.04	−0.06 ± 0.10	6.78 ***	−0.34 ± 0.36	0.13 ± 0.10	3.13
	FC3	−0.35 ± 0.08	0.41 ± 0.04	21.32 ***	0.00 ± 0.16	−0.12 ± 0.07	1.61
	FC4	−0.68, ± 0.15	0.00 ± 0.07	10.04 ***	−0.61 ± 0.21	−0.20 ± 0.12	4.33 **
	Cz	−0.03 ± 0.03	0.72 ± 0.11	16.00 ***	−0.11 ± 0.08	0.47 ± 0.20	6.55 ***
	Pz	0.38 ± 0.06	1.23 ± 0.14	13.64 ***	−0.03 ± 0.07	0.64 ± 0.24	6.51 ***
	Oz	−0.24 ± 0.07	0.29 ± 0.10	10.50 ***	−0.21 ± 0.09	0.09 ± 0.18	3.52 *
Unpleasant	Fz	−0.13 ± 0.20	0.97 ± 0.07	12.96 ***	0.08 ± 0.09	−0.07 ± 0.21	1.62
	F3	0.33 ± 0.16	1.29 ± 0.07	13.42 ***	0.52 ± 0.22	0.49 ± 0.32	0.11
	F4	0.30 ± 0.10	0.89 ± 0.07	12.12 ***	0.04 ± 0.12	0.14 ± 0. 29	0.75
	FC3	0.49 ± 071	0.42 ± 0.08	1.52	−0.27 ± 0.20	−0.30 ± 0.05	0.33
	FC4	0.59 ± 0.08	0.09 ± 0.13	7.94 ***	0.34 ± 0.27	−0.44 ± 0.24	5.22 ***
	Cz	0.51 ± 0.15	1.77 ± 0.11	16.16 ***	0.16 ± 0.20	−0.07 ± 0.32	1.46
	Pz	0.21 ± 0.13	1.71 ± 0.76	4.73	−0.44 ± 0.16	−0.02 ± 0.28	3.22 *
	Oz	0.58 ± 0.15	1.60 ± 0.09	13.84 ***	0.38 ± 0.21	−0.18 ± 0.29	3.82 **

Note: * *p* < 0.028 average family false discovery rate threshold, ** *p* < 0.01, *** *p* < 0.001.

## Data Availability

Data are available upon request to A.D.
